# Upregulation of TRESK Channels Contributes to Motor and Sensory Recovery after Spinal Cord Injury

**DOI:** 10.3390/ijms21238997

**Published:** 2020-11-26

**Authors:** Gyu-Tae Kim, Adrian S. Siregar, Eun-Jin Kim, Eun-Shin Lee, Marie Merci Nyiramana, Min Seok Woo, Young-Sool Hah, Jaehee Han, Dawon Kang

**Affiliations:** 1 Department of Physiology and Institute of Health Sciences, College of Medicine, Gyeongsang National University, Jinju 52727, Korea; gtkim3779@naver.com (G.-T.K.); adriansiregar46@gmail.com (A.S.S.); eunjin1981@hanmail.net (E.-J.K.); mariemerci1994@naver.com (M.M.N.); whitewms@naver.com (M.S.W.); jheehan@gnu.ac.kr (J.H.); 2 Department of Convergence Medical Science, Gyeongsang National University, Jinju 52727, Korea; 3 Department of Rehabilitation Medicine, College of Medicine, Gyeongsang National University, Jinju 52727, Korea; rmeslee@gnu.ac.kr; 4 Biomedical Research Institute, Gyeongsang National University Hospital, Jinju 52727, Korea; imhappyto@hanmail.net

**Keywords:** dorsal root ganglion, inflammation, oxidative stress, spinal cord injuries, two-pore domain K^+^ channel

## Abstract

TWIK (tandem-pore domain weak inward rectifying K^+^)-related spinal cord K^+^ channel (TRESK), a member of the two-pore domain K^+^ channel family, is abundantly expressed in dorsal root ganglion (DRG) neurons. It is well documented that TRESK expression is changed in several models of peripheral nerve injury, resulting in a shift in sensory neuron excitability. However, the role of TRESK in the model of spinal cord injury (SCI) has not been fully understood. This study investigates the role of TRESK in a thoracic spinal cord contusion model, and in transgenic mice overexpressed with the TRESK gene (TG_TRESK_). Immunostaining analysis showed that TRESK was expressed in the dorsal and ventral neurons of the spinal cord. The TRESK expression was increased by SCI in both dorsal and ventral neurons. TRESK mRNA expression was upregulated in the spinal cord and DRG isolated from the ninth thoracic (T9) spinal cord contusion rats. The expression was significantly upregulated in the spinal cord below the injury site at acute time points (6, 24, and 48 h) after SCI (*p* < 0.05). In addition, TRESK expression was markedly increased in DRGs below and adjacent to the injury site. TRESK was expressed in inflammatory cells. In addition, the number and fluorescence intensity of TRESK-positive neurons increased in the dorsal and ventral horns of the spinal cord after SCI. TG_TRESK_ SCI mice showed faster paralysis recovery and higher mechanical threshold compared to wild-type (WT)-SCI mice. TG_TRESK_ mice showed lower TNF-α concentrations in the blood than WT mice. In addition, IL-1β concentration and apoptotic signals in the caudal spinal cord and DRG were significantly decreased in TG_TRESK_ SCI mice compared to WT-SCI mice (*p* < 0.05). These results indicate that TRESK upregulated following SCI contributes to the recovery of paralysis and mechanical pain threshold by suppressing the excitability of motor and sensory neurons and inflammatory and apoptotic processes.

## 1. Introduction

Spinal cord injuries (SCIs) result in many biomedical and molecular changes. A primary injury, which consists of severed axons, dying neurons, and glia, and a disturbed microvasculature, triggers a cascade of pathological events, including vascular and biochemical changes, free radical formation, local acidosis, hemorrhagic necrosis, and inflammatory processes [1,2,3,4]. These changes result in severe motor and sensory dysfunctions, such as paralysis and pain, which is a serious and common problem after an SCI [5]. Therefore, SCI animal models have been used to determine mechanisms of motor dysfunctions and pain [5]. These animal models show both sensory and motor dysfunction [6,7]. The spinal contusion is the oldest and most widely used SCI animal model.

SCIs trigger an interactive reaction of anatomical, neurochemical, excitotoxic, and inflammatory properties, resulting in neuronal hyperexcitability. Spontaneous electrical activity can arise at the site of nerve injury and in neuronal cell bodies of dorsal root ganglia (DRGs) [8], which is a novel target for neuromodulation [9]. The excitability of DRG neurons is regulated in response to a number of pathological conditions [10]. Abnormal excitation of injured neurons is attributed to alterations in the expression and functional characteristics of many kinds of channels and transporters, such as Na^+^, Ca^2+^, K^+^, and Cl^−^ [11,12]. Among them, K^+^ channels play an important role in determining the electrical properties of DRG neurons. DRG neurons express multiple types of K^+^ channels, including voltage-dependent (K_v_), inwardly rectifying (K_ir_), Ca^2+^-activated (K_Ca_), and two-pore domain K^+^ (K_2P_) channels [13].

The background K^+^ conductance in DRG neurons is composed of currents provided by the members of the K_2P_ channel family. The mRNA transcripts of at least eight K_2P_ channels are expressed in DRG neurons [14,15]. TWIK (tandem-pore domain weak inward rectifying K^+^)-related spinal cord K^+^ channel (TRESK), which is the most recently cloned K_2P_ channel, is abundantly expressed in rat DRG neurons [15,16]. TRESK is mainly expressed in small to medium diameter sensory neurons, corresponding to nociceptive neurons [16]. In particular, TRESK is predominantly expressed in non-peptidergic IB4^+^ sensory neurons, but not in peptidergic sensory neurons, suggesting involvement in neuropathic pain [17]. Numerous studies have demonstrated that TRESK is involved in the regulation of excitability of specific subtypes of sensory neurons [18]. In addition, it is well documented that TRESK expression is downregulated in several models of peripheral nerve injury, resulting in a shift in sensory neuron excitability [18]. However, changes in expression and function of TRESK in an SCI have not been fully understood.

This study was performed to identify the role of TRESK in a thoracic spinal cord contusion model. In addition, transgenic mice overexpressed with the TRESK gene (TG_TRESK_) were used to further understand the role of TRESK.

## 2. Results

### 2.1. TRESK Expression in DRG Neurons

**Figure 1:**
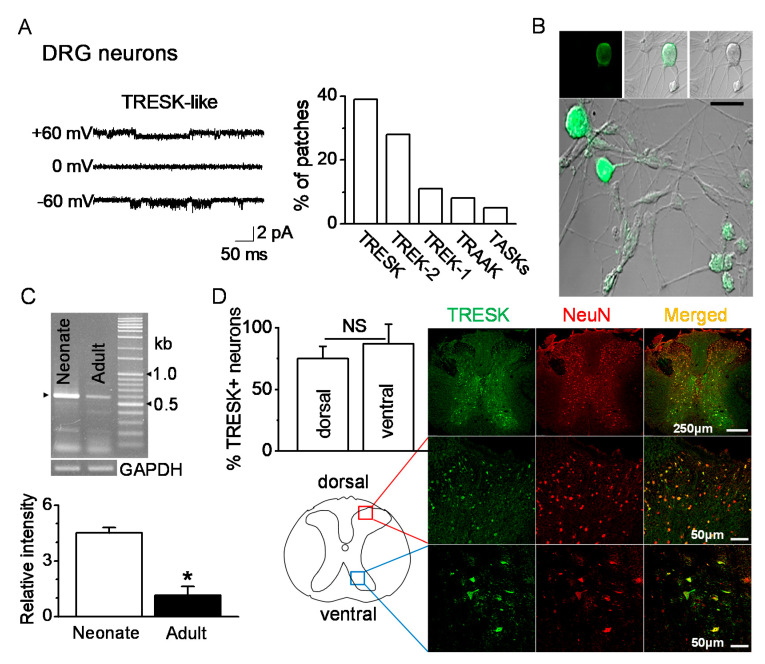
Expression of TRESK (tandem-pore domain weak inward rectifying K^+^ (TWIK)-related spinal cord K^+^ channel) in dorsal and ventral neurons. (**A**) Functional expression of TRESK in DRG neurons isolated from neonate (P1-2) rats by single-channel recording. Representative traces show TRESK channel current at +60 mV (upper trace), 0 mV (middle), and −60 mV (lower). Pipette and bath solutions contained 150 mM KCl. The bar graph summarizes the percentage of patches showing each of the five K_2P_. (**B**) Immunocytochemical analysis of TRESK in DRG neurons of various diameters. Fluorescent images labeled with TRESK-specific antibody and fluorescein isothiocyanate (FITC)-conjugated anti-rabbit IgG. (left panel, fluorescent images; right panel, differential interference contrast (DIC) images). Middle and lower panels show merged images of green fluorescence and DIC images. Scale bar represents 30 μm. (**C**) Changes in TRESK mRNA expression in DRG with age. In neonate (P1-2) and adult (P120) DRGs, the TRESK mRNA PCR product (578 bp) was obtained and confirmed by sequencing. Each bar represents mean ± SD of three independent experiments. * *p* < 0.05 compared to neonate. (**D**) Expression of TRESK in the dorsal and ventral horns of the thoracic spinal cord of adult mice. Representative images of TRESK (green) expressed in neurons marked by NeuN (red). The images were taken from the deparaffinized tissue section. The bar graph shows the percentage of TRESK-positive cells in the NeuN-positive cells of the dorsal and ventral horns. NS, non-significant.

Single-channel recordings were performed in postnatal rat day 1 or day 2 (P1-2) DRG neurons ranging from 10 to 38 μm in diameter. TRESK-like channels in DRG neurons were functionally expressed. At +60 and −60 mV, the single-channel conductances of the TRESK-like channels were 14 pS and 15 pS, respectively (Figure 1A). TRESK-like channels were observed in many patches (39%, 47 of 120 cell-attached patches studied) at room temperature. Immunocytochemistry data showed TRESK expression in P1 DRG neurons of various diameters (Figure 1B). RT-PCR data showed expression of TRESK mRNA in DRG. The TRESK mRNA expression changed with age. TRESK mRNA expression levels were significantly decreased in adult DRG (P120) by 80% compared to neonatal DRG (P1-2) (*n* = 5, *p* < 0.05, Figure 1C). TRESK expression patterns were analyzed in the spinal dorsal and ventral horns of the adult mice. TRESK was expressed in NeuN-labeled neurons in the dorsal and ventral horns. There was no significant difference in the percentage of TRESK-positive neurons between the dorsal and ventral horns (*n* = 4, *p* > 0.05, Figure 1D).

### 2.2. Changes in TRESK mRNA Expression after Spinal Cord Injury

**Figure 2:**
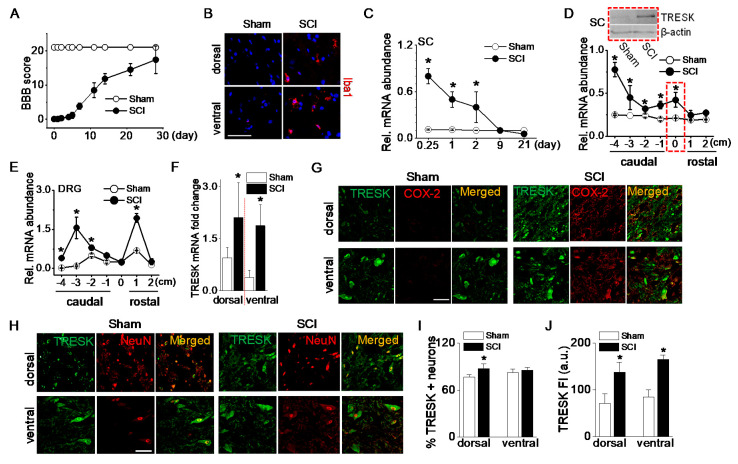
Upregulation of TRESK expression after spinal cord injury. (**A**) The BBB open-field locomotor rating scores showed spontaneous partial recovery of motor function after spinal cord injury (SCI; *n* = 20 animals per group). Data points represent mean ± SD. (**B**) Iba1-positive cells detected in spinal cord of the SCI group. Representative images of Iba1 (red) expression and DAPI staining for nucleus (blue). The images were taken from the frozen tissue section. (**C**) Alteration in TRESK mRNA expression at various times after an SCI. First strand cDNAs were synthesized from total RNA isolated from the spinal cord at the injury site. The relative mRNA levels were analyzed by real-time PCR and normalized to GAPDH. Data are expressed as the mean ± SD (*n* = 8). (**D**) Alteration in TRESK mRNA expression in the spinal cord at, above (rostal), and below (caudal) the lesion. Data are expressed as the mean ± SD (*n* = 8). Upper blot showed an increase in TRESK protein in the spinal cord at the lesion (T9). The dotted boxes represent results obtained from T9 lesions. The +, 0, and − indicate above the injury (rostral), at the injury, and below the injury level (caudal), respectively. The 1 and 4 indicate adjacent to and far from the injury site, respectively. (**E**) Alteration in TRESK mRNA expression in the DRG at, above, and below the lesion. Data are expressed as the mean ± SD (*n* = 5). All results were normalized to the expression of GAPDH mRNA. (**F**) TRESK mRNA fold change in the dorsal and ventral horn of sham and the SCI group. Data are expressed as the mean ± SD (*n* = 4). (**G**) TRESK expression in inflammatory cells increased in the dorsal and ventral horns of the thoracic spinal cord after the SCI. Cells expressing both TRESK (green) and COX-2 (red) are shown in yellow. (**H**) Increase in TRESK expression level in the SCI group. The merged images of TRESK (green) and NeuN (red) expression are shown in yellow. (**I**,**J**) Bar graphs show the percentage of TRESK-positive neurons and TRESK fluorescence intensity (FI) in the dorsal and ventral horns. * *p* < 0.05 compared to each corresponding control. SC and DRG represent the spinal cord and dorsal root ganglion, respectively. The au represents an arbitrary unit. Scale bar, 50 μm.

Changes in TRESK mRNA and protein expression were analyzed in adult rats following a 25-mm contusion to the ninth thoracic (T9) spinal cord segment. Open field motor function was assessed after injury using the Basso-Beattie-Bresnahan (BBB) locomotor rating scale, which assesses the degree of recovery of hind limb function after spinal cord contusion [19]. Following an SCI, rats initially showed paralysis, but they spontaneously recovered movement of both hind limbs as time passed. The average BBB score was 16 ± 3 at 28 days (Figure 2A). Microglial activation in the spinal cord after an SCI was investigated as one of the inflammatory responses. Ionized calcium-binding adapter molecule 1 (Iba1), a marker of microglial activation, positive cells were more observed in the spinal dorsal and ventral horns of the SCI group compared to the sham-operated group (Figure 2B). To examine the time-dependent changes in TRESK expression level following an SCI, spinal cords were harvested at varying time points between 6 h and 21 days after the SCI. TRESK mRNA expression was significantly upregulated in the spinal cord at the injury site at acute points (6, 24, and 48 h) after an SCI (*p* < 0.05, Figure 2C). Further experiments focused on the samples harvested at 48 h after an SCI. At this time, the anesthetic effect disappeared, and the degree of inflammation was high.

TRESK mRNA levels in the spinal cord were significantly increased at the injury site and below the injury (*p* < 0.05, Figure 2D). In particular, TRESK was dramatically upregulated in the far caudal segment of the cord (−4). TRESK protein levels were also markedly increased in the injured spinal cord of SCI rats (Figure 2D blot).

To identify whether TRESK expression in DRG is changed at sites above- or below- the injury, a further study was performed using rostral and caudal DRG. Seven segments of DRG (1 cm long each) were isolated: One centered at the injury site (within 1 mm of the injury center), two were rostral, and four were caudal. TRESK mRNA expression was upregulated in rostral and caudal segments of DRG. As shown in Figure 2E, TRESK was increased in DRG adjacent to the injury site (+1) and caudal segments (−2, −3, and −4). There was no significant difference in DRG isolated from the injury site (0) of sham and SCI groups. TRESK mRNA expression level was analyzed in dorsal and ventral horns of the spinal cord. In the SCI group, TRESK mRNA expression levels were significantly high in both dorsal and ventral horns compared to the sham group (*n* = 4, *p* < 0.05, Figure 2F).

The number of cells expressing cyclooxygenase (COX)-2, which is expressed in inflammatory cells, was higher in the SCI group. TRESK was expressed in inflammatory cells expressing COX-2 in both dorsal and ventral horns (Figure 2G). The number and fluorescence intensity of TRESK-positive neurons increased in the dorsal and ventral horns of the spinal cord after an SCI (Figure 2H). The percentage of TRESK-positive neurons increased significantly in the dorsal horn after an SCI (*n* = 5, *p* < 0.05, Figure 2I), and the fluorescence intensity of TRESK-positive neurons increased significantly in both the dorsal and ventral horns of the SCI group compared to the sham group (Figure 2J).

### 2.3. Role of TRESK on Sensory and Motor Recovery from an SCI

To further understand the role of TRESK in a T9 spinal cord segment contusion model, transgenic mice overexpressed with the TRESK gene (TG_TRESK_) were generated (see Supplementary Figure S1A–C). TG_TRESK_ mice displayed a normal behavioral phenotype, and their genotype was confirmed by PCR. All tissues tested (cerebrum, cerebellum, spinal cord, DRG, thymus, spleen, testis, ovary, heart, kidney, liver, lung, stomach, and muscle) expressed TRESK mRNA (Supplementary Figure S1D). The mRNA expression level was normalized to GAPDH. In TG_TRESK_ mice, TRESK mRNA levels were significantly increased in all tissues tested, except stomach and ovary, compared to wild-type (WT) mice (Supplementary Figure S1E, *n* = 5, *p* < 0.05).

**Figure 3:**
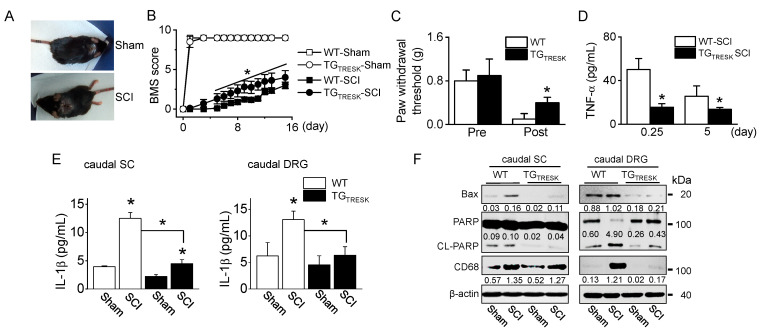
Motor and sensory recovery in T9 injured wild-type (WT) and TG_TRESK_ mice. (**A**) Photographs of mice with a T9 spinal segment injury. The injured mice exhibited paralysis of the hind limbs (SCI). (**B**) The BMS score of four groups (wild-type sham and SCI and TG_TRESK_ sham and SCI). Data are expressed as the mean ± SD of 20 animals per each group. (**C**) Reduction of mechanical allodynia following TRESK upregulation. The von Frey filaments were applied to the hind limb paw, and the threshold values to hind limb paw withdrawal were measured. Data are expressed as the mean ± SD of ten animals per each group. (**D**) Plasma TNF-α concentration in serum of WT-SCI and TG_TRESK_ SCI mice. Data are expressed as the mean ± SD (*n* = 10). (**E**) The decrease in IL-1β concentration in the caudal spinal cord and DRG tissues obtained from TG_TRESK_ mice. Data are expressed as the mean ± SD (*n* = 4). * *p* < 0.05 compared to each corresponding control (**B**–**E**). (**F**) The decrease in apoptotic and inflammatory proteins in the caudal spinal cord and DRG tissues obtained from TG_TRESK_ mice. Western blot analysis of pro-apoptotic Bax, cleaved PARP, and CD68. Cell lysate (30 μg of protein) was loaded on gel lane for immunoblotting. The number between blots represents the normalized ratio of the Bax and CD68 protein level to β-actin protein level for each lane. CL-PARP level was normalized to total PARP level.

T9 SCI animal model was generated using TG_TRESK_ mice. After T9 SCI, all mice showed paralysis of both hind limbs (Figure 3A), corresponding to a BBB score of 0. The BMS score was measured for 15 days. On the day after the SCI, the BMS scores markedly decreased in all groups, but they gradually increased in both sham groups. TG_TRESK_ SCI mice showed more rapid relief than WT-SCI mice in motor behavior, as judged by the BMS open-field locomotor rating scores (*n* = 20 animals per each group, *p* < 0.05, Figure 3B). In the WT-SCI model, the von Frey test showed incremental mechanical allodynia in the hind paws with a reduction in the threshold of paw withdrawal to stimulation from 0.8 g before the SCI to 0.1 g following the SCI. In TG_TRESK_ SCI mice, the mechanical threshold was high compared to that of WT-SCI mice (Figure 3C). In SCI models, TG_TRESK_ mice showed lower tumor necrosis factor (TNF)-α plasma concentration than WT mice (*n* = 12, Figure 3D). In the caudal spinal cord and DRG, inflammatory and apoptotic proteins were identified. Interleukin (IL)-1β, a pro-inflammatory cytokine, significantly increased in SCI mice compared to sham mice, whereas the IL-1β concentration was significantly decreased in TG_TRESK_ SCI mice compared to WT-SCI mice (*n* = 4, *p* < 0.05, Figure 3E). Bcl-2-associated X protein (Bax) and cleaved poly (ADP-ribose) polymerase (PARP), apoptotic proteins, and a cluster of differentiation 68 (CD68), a marker of inflammation associated with monocytes/macrophages infiltration, were upregulated in SCI mice. However, the expression levels were reduced in TG_TRESK_ SCI mice. TG_TRESK_ sham mice showed low expression of Bax, cleaved PARP (CL-PARP), and CD68 in the caudal spinal cord and DRG (*n* = 3, Figure 3F).

## 3. Discussion

Injury is accompanied by the firing of quiescent neurons and hyperexcitation of DRG neurons. Hyperexcitation has been attributed to activation of Na^+^ and nonselective cation channels and inhibition of K^+^ channel. K_2P_ channel is constitutively open, so it is involved in setting the resting membrane potential by generating a background K^+^ current in neurons. Thus, inhibition of background K^+^ currents increases neuronal excitability [18]. In DRG neurons, TRESK is a main background K^+^ current [16]. Numerous studies have demonstrated that TRESK reduction enhances the excitability of nociceptive neurons [18], and that TRESK overexpression reduces the excitability of nociceptive neurons and pain sensitivity in animal models [20,21]. On the other hand, in TRESK global knockout (KO) mice, trigeminal ganglion neurons show hyperexcitation compared to WT mice [22,23], but lumbar DRG neurons do not show hyperexcitation [22]. Another previous study reports that TRESK KO mice generated by random mutagenesis (G339R) inactivating the second selectivity filter show an increase in DRG neuronal excitability, but do not affect resting membrane potentials [24], suggesting that TRESK contributes to the regulation of cellular excitability rather than the control of the resting membrane potential of DRG neurons. In addition, the expression of TRESK can be expressed differently in the body regions in the peripheral nerve injury model or in the KO model, and the moderate phenotypic consequences in TRESK KO mice can result from the compensatory replacement of channels by closely related members of the K_2P_ family expressed in sensory neurons. In line with previous studies, the TG_TRESK_ mice generated in this study showed more significant rapid movement in the open-field test and lower sensitivity in response to mechanical stimuli using van Frey filaments compared to WT mice.

The thoracic spinal cord contusion model is the most commonly used for SCI neuropathic pain research [25]. A SCI often causes neuropathic pain, which is difficult to treat, due to complex pathology and various patterns. Neuropathic pain has been classified into at-level, above-level, and below-level pains. In thoracic SCI, trunk, forelimb, and hind limb allodynia indicate at-level, above-level, and below-level neuropathic pain, respectively [26]. In addition, thoracic SCI has been reported to cause an increased generation of spontaneous activity combined with hyperexcitability in DRG sensory neurons below the injury site [27]. In the mechanical pain test using von Frey filaments, TG_TRESK_ T9 SCI showed lower sensitivity in the hind limb than WT SCI mice, suggesting that TRESK may be involved in the reduction of SCI-induced below-level neuropathic pain. After SCI, TRESK expression was increased in DRG of below-level rather than the injured region. TRESK upregulation in the caudal region is likely to be at least partially responsible for the reduction in below-level pain sensation and facilitated behavioral relief.

In experimental animal models for peripheral neuropathic pain, TRESK expression was highly decreased in DRGs after injury [21,28,29]. However, strictly speaking, the injured animal model and the region are different among studies. Hwang et al., (2015) reported an increase in TRESK expression in the dorsal horn of the spine after L5–6 spinal nerve ligation, but a decrease in L5 DRG [30]. In a sciatic nerve cuffing-induced neuropathic pain model, TRESK KO mice had a lower mechanical threshold 5 to 7 days after injury, but not at the 14 and 21 post-injury days [31]. In the T9 SCI model, we found that there was no change in TRESK expression in T9 DRG, but it was upregulated at a below level than the injury site. In addition, TRESK was highly upregulated in the sensory and motor neurons of the spinal cord below the level of injury. The expression pattern dramatically appeared in the spinal cord at acute time points (6, 24, and 48 h) after the SCI. It is difficult to directly compare changes in TRESK expression between the peripheral nerve injury model and the T9 SCI model, as the injury site is completely different and the damaging effect could be different. In the current situation, we do not know exactly how the expression of TRESK varies with an injury. However, we suggest that TRESK may act as a protective mediator against the SCI and neuropathic pain as it depends on the severity, region, and timing of the SCI.

Following an SCI, pathophysiology arises from both primary and secondary mechanisms. Pathological conditions triggered by SCIs show as an increase in free radical formation, calcium overload, and excessive glutamate accumulation [32], which activate TRESK [33,34]. On the other hand, SCI-induced acidosis and an increase in free fatty acids could suppress TRESK activity [35]. These complex conditions seem to be able to regulate TRESK expression and activity. TRESK modulation by these factors could be a key for the control of cell excitability, K^+^ homeostasis, and resting membrane potential in the spinal cord and DRG neurons. Herein, we suggest that TRESK can counteract SCI-induced pathological conditions, as shown by the protective effect of TRESK on inflammation and apoptosis. Mice overexpressing TRESK would also be able to demonstrate a facilitated relief effect from an SCI and lower pain sensitivity. In addition, upregulation of TASK-3 following an SCI [36] can help the relieving effect of TRESK from an SCI by controlling cellular excitability and resting membrane potential. Ionic disorders, among the many aspects of the SCI, cause multifaceted pathophysiological effects. A combination therapy based on ion channel modulation may be an effective treatment for the SCI. TRESK is regulated by a variety of factors, such as pH, free fatty acid, Ca^2+^, GPCR agonists, analgesics, and antidepressants. Therefore, TRESK may be an important target for the development of analgesic and anti-inflammatory drugs that can be used to treat the SCI. In addition, it is thought that TRESK activators and upregulators have the potential to be used for the treatment of the SCI and neuropathic pain.

The limitations of this study were that we did not measure changes in membrane potential and excitability in motor and sensory neurons in the WT and TG_TRESK_ sham and SCI models. Also, we did not differentiate subtypes of cells expressing TRESK. The association between the level of TRESK expression and its protective role associated with post-SCI recovery should be more accurately determined by analyzing the excitatory changes in TG_TRESK_ mice. In addition, several appropriate behavioral tests are needed to assess TRESK-related motor and sensory recovery after an SCI.

## 4. Materials and Methods

### 4.1. Animal Care

Sprague-Dawley rats and C57BL/6 mice were purchased from Koatech Co. (Animal Breeding Center, Korea). Animal experiments were conducted in accordance with the guidelines of the National Institute of Health Guide for the Care and Use of Laboratory Animals (NIH Publication No. 80-23) and the Gyeongsang National University Animal Care and Use Committee (GLA-090805-R0065 and GLA-100310-M0024). Animals were housed under a 12-h light/dark cycle in pathogen-free conditions, with food and water freely available. All experiments were performed with the approval of the Research Ethics Committee of Gyeongsang National University.

### 4.2. Generation of TRESK Transgenic Mice

TG_TRESK_ mice (C57BL/6) were generated by Macrogen’s mouse division (Macrogen Inc, Seoul, Korea). TRESK (GenBank accession number, NM_207261) was expressed in the pcDNA3.1/V5-His-TOPO vector and was restricted by three enzymes (*Nru*I, *Dra*III, and *Sca*I). The DNA fragment was used as a transgene for microinjection into the male pronucleus of zygotes superovulated by an intraperitoneal injection of seven IU of pregnant mare serum gonadotrophin (PMSG), followed by an injection of seven IU of hCG 48 h. A TRESK gene containing the CMV sequence was detected by PCR. Homozygous TG_TRESK_ mice used in this study were bred from F3 to F5 heterozygotes. TG_TRESK_ mice were housed in Gyeongsang National University Animal Center.

### 4.3. Spinal Cord Contusion Injury Animals

Following an SCI, animals suffer from neurogenic voiding dysfunction. Thus, female animals were used for efficient care and maintenance after surgery, because they have shorter urethras than males. Rats (250 g, 12 weeks old, *n* = 60) and mice (8 weeks old, *n* = 60) were deeply anesthetized by intraperitoneal injection with 3 mg/kg Rompun^®^ (Bayer Korea, Seoul, Korea) and Zoletil50^®^ (Virbac, Carros, France). The skin was incised along the midline of the back, and the paravertebral muscles of the thoracic-level (T8–T10) vertebrae were dissected out. Forty rats were subjected to an SCI at the ninth thoracic spinal cord segment (T9) under visual guidance using the New York University (NYU) Impactor (New York, NY, USA). A 2.0-mm diameter rod was released from a 25-mm height onto the exposed spinal cord. An acute-compression SCI was inflicted in mice by a one minute extradural compression at T9 using a vascular clamp (with 30 g force, S&T AG, Neuhausen, Switzerland) under visualization using a stereozoom microscope (SZ60, Olympus, Tokyo, Japan). Sham-operation animals (*n* = 20) underwent the same procedures as the SCI animals, except for the contusion or compression. After the SCI or sham surgery, the overlying muscles and skin were closed in layers with 4.0 silk sutures, and the animals were allowed to recover on a heating pad. They were then returned to their home cages, with free access to food and water. Following the operation, Fortecillin comp (penicillin streptomycin HCl, Bayer Korea, Seoul, Korea) was administered twice a day at a dose of 20,000 IU/kg to prevent urinary tract infection. Bladders were manually emptied twice per day until the bladder-emptying reflex returned (typically within nine days after the injury). TG_TRESK_ mice were also subjected to the same SCI operation.

### 4.4. Basso-Beattie-Bresnahan (BBB) and Basso Mouse Scale (BMS) Locomotor Scoring

A behavioral test, the BBB test [19] was performed on rats to measure the recovery of hindlimb function after spinal cord contusion. Briefly, rats were adapted to the open field (1.5 × 0.6 m) used for the test were by allowing them to walk freely in the open field for 4 min. Hindlimb function was assigned a score ranging from a minimum of 0 (no observable movement) to a maximum of 21 (normal locomotion). The test was performed for four weeks post-SCI.

For mice, the BMS open-field test was used [37]. Briefly, the BMS uses a 9-point scale that provides a gross indication of locomotor ability and determines the phases of locomotor recovery and features of locomotion. During the early phase of recovery, resolution of paralysis and/or paresis progressed from no ankle movement to more pronounced ankle movement, which was related to a score of 0–2. Plantar placing and stepping occur in the intermediate phase of recovery, which earns a score of 3–4. In the late phase of recovery, paw position during stance, hindlimb-forelimb coordination, and trunk stability were analyzed, resulting in a score of 5–8. A score of 9 indicates normal locomotor mobility, with trunk stability and refined performance. The score for each animal was recorded as the average of the left and right hind limbs to obtain one BMS score per mouse, and then the mean of the group was calculated. For these tests, the animals were evaluated before the injury, 24 h after surgery, and then weekly up to 8 weeks post-injury.

### 4.5. Mechanical Allodynia

The mice were placed in a transparent plexiglass cubicle testing apparatus on a metal mesh floor and allowed to acclimate for 30 min. All mice were tested to determine the withdrawal threshold of the hind paw in response to the von Frey filaments (Stoelting, Wood Dale, IL, USA) using the up-down method [38] using a logarithmic series of calibrated monofilaments with bending forces ranging 0.1 to 10 g. The von Frey filaments were applied perpendicularly to the mid-plantar surface of both hind paws with sufficient force and were pressed to the point of bending for 1–2 s. Rapid withdrawal of the stimulated paw was considered a positive response. The threshold force required to elicit withdrawal of the paw was determined twice for each hind paw on each testing day, with sequential measurements separated by at least 5 min. Filaments were applied six times in the order of increasing stiffness. The first filament that evoked at least one response was recorded as the threshold. Mechanical allodynia was determined by a significant decrease in the mechanical threshold of T9 injured mice compared to sham-operated mice.

### 4.6. Isolation of Dorsal Root Ganglion (DRG) and Culture of DRG Neurons

DRG adjacent to the T9 were dissected from sham-operated and SCI animals. Cultured DRG neurons were prepared as described previously [39]. Briefly, DRG were dissected from both levels of the thoracic and lumbar spinal cord of 21 neonatal rats (postnatal day 1 or day 2, P1-2) and collected in cold (4 °C) Dulbecco’s modified Eagles’ medium (DMEM) containing 10% fetal bovine serum (FBS; Invitrogen, Grand Island, NY, USA), 1 mM sodium pyruvate, 25 ng/mL nerve growth factor, 10 U/mL penicillin (Invitrogen), and 10 μg/mL streptomycin (Invitrogen). Ganglia were washed three times with DMEM and incubated for 30 min in DMEM containing 1 mg/mL Type II collagenase (Worthington, Freehold, NJ, USA). Ganglia were then washed three times with Mg^2+^- and Ca^2+^-free Hank’s Balanced Salt Solution (HBSS) and were incubated with gentle shaking in warm (37 °C) HBSS containing 2.5 mg/mL trypsin (Invitrogen). The solution was centrifuged at 1,000 rpm (194× *g*) for 10 min, and the pellet was washed three times with DMEM containing 10% FBS to inactivate the enzyme. The pellet was suspended in a culture medium and gently triturated with a heat-polished Pasteur pipette. The suspension was plated on glass coverslips coated with poly-l-lysine and placed in a culture dish. DRG neurons were incubated at 37 °C in a humidified atmosphere of 95% air and 5% CO_2_. DRG neurons were used 1–5 days after plating, and the medium was replaced every two days.

### 4.7. Reverse Transcriptase-Polymerase Chain Reaction (RT-PCR) Analysis

Total RNA was isolated from RAW264.7 cells, DRGs, spinal cord, and other organs of control, sham, SCI, and TG_TRESK_ using TRIzol Reagent (Invitrogen). First-strand cDNAs were synthesized from total RNA isolated from each organ using oligo (dT) (RT-&GO Mastermix, Qbiogene, Cambridge, UK) and then used as a template for PCR amplification. Specific primers for TRESK were used in PCR reactions with *Taq* polymerase (G-Taq^TM^, Cosmo Genetech, Seoul, Korea). Table 1 lists the DNA sequences of the primers used to detect the expression of TRESK and pro-inflammatory mediators. PCR was conducted in a final reaction volume of 30 μL containing 1 μL (~50 ng) of diluted first-strand cDNA. PCR conditions included an initial denaturation at 94 °C for 5 min, followed by 30 cycles at 94 °C for 30 s, 60 °C for 45 s, and 72 °C for 45 s, and a final extension step at 72 °C for 10 min. The PCR products were directly sequenced with the ABI PRISM^®^ 3100-Avant Genetic Analyzer (Applied Biosystems, Foster City, CA, USA).

### 4.8. Real-Time PCR Analysis

Changes in TRESK mRNA expression in the brain, spinal cord, and DRG following the SCI were quantified using real-time PCR with FastStart DNA Master SYBR Green I (Roche Applied Science, Mannheim, Germany) and the LightCycler System (LightCycler 2.0 instrument, Roche). Real-time PCR primers were designed using GenScript Online PCR Primers Designs Tool (GenScript, Piscataway, NJ, USA). PCR conditions consisted of a denaturing cycle (95 °C for 10 min), 40 cycles of PCR (95 °C for 7 s, 56 °C for 7 s, and 72 °C for 10 s), a melting cycle (65 °C for 60 s), a step cycle (increase from 65 °C to 95 °C at a rate of 0.1 °C/s), and a cooling step (40 °C for 30 s). Melt-curve analysis was conducted to confirm that each product was produced, and the correct product size was confirmed on a 1.5% agarose gel. The 2^−ΔΔCT^ method was used to calculate the relative levels of TRESK mRNA [40]. Relative TRESK mRNA abundance level was normalized to the glyceraldehyde-3-phosphate dehydrogenase (*GAPDH*) level.

### 4.9. Western Blot Analysis

Rodent DRG and spinal cord were homogenized in lysis buffer containing 50 mM HEPES (pH = 7.5), 150 mM NaCl, 10% glycerol, 100 mM NaF, 0.2 mM NaVO_3_, 0.5% NP-40, 1.5 mM MgCl_2_, 1 mM EGTA, 1 mM dithiothreitol (DTT), 1 g/mL leupeptin, 10 mM benzamidine, 1 g/mL pepstatin A, 1 mM phenylmethylsulfonyl fluoride (PMSF), and 10.5 g/mL aprotinin. These tissues were then incubated for 30 min on ice with intermittent vortexing. Extracts were clarified by centrifugation at 14,000 rpm (19,300× *g*) for 20 min at 4 °C. The resulting supernatant was subjected to a 10% SDS-polyacrylamide gel and separated by electrophoresis for 120 min at 120 V. Then, the gel was transferred to a polyvinylidene difluoride (PVDF) membrane (Millipore, Billerica, MA, USA) for 1 h at 100 V using a wet transfer system (Bio-Rad, Hercules, CA, USA). The membranes were blocked with 5% fat-free dry milk and incubated with TRESK polyclonal antibody (1:500 dilution, Alomone Labs, Jerusalem, Israel/Santa Cruz Biotechnology, Santa Cruz, CA, USA), anti-Bax (1:200 dilution, Santa Cruz Biotechnology), anti-CD68 (1:200 dilution, Santa Cruz Biotechnology), anti-PARP (1:1,000 dilution; Cell Signaling, Danvers, MA, USA), and anti-β-actin antibody (1:5000 dilution; Thermo Fisher Scientific, Rockford, IL, USA) at 4 °C overnight. The primary antibody incubation was followed by incubation with a secondary peroxidase-conjugated anti-rabbit or anti-mouse antibody at 1:5000 (Assay designs, Ann Arbor, MI, USA). Immuno-positive bands were visualized by enhanced chemiluminescence (SuperSignal^TM^ WestPicoPLUS Luminol/Enhancer, Thermo Fisher Scientific) using the iBright^TM^ CL1500 imaging system (Thermo Scientific Fisher/Life Technologies Holdings Pte Ltd., Singapore).

### 4.10. Immunostaining

DRG neurons were fixed with 4% paraformaldehyde in 0.1 M phosphate-buffered saline (PBS). The DRG neurons cultured on the coverslips were washed with PBS and incubated in a blocking buffer containing 1% normal goat serum and 0.1% Triton X-100 in PBS for 2 h at room temperature. Deparaffinized and frozen spinal cord sections were subjected to immunostaining. Deparaffinized tissue sections were permeabilized with 0.2% Triton X-100 for 10 min at room temperature. Frozen tissue sections were fixed with 4% paraformaldehyde for 10 min at room temperature. After washing three times in PBS, all sections were incubated in a blocking buffer containing 1.5% normal goat serum in PBS for 1 h at room temperature. After blocking, the cells and tissue sections were incubated overnight with affinity-purified polyclonal antibodies directed against the TRESK proteins (Alomone Labs) diluted 1:200 in PBS, anti-Iba1 (1:200 dilution, Abcam, Cambridge, UK), anti-COX-2 (1:50 dilution, Santa Cruz Biotechnology), and anti-NeuN (1:200 dilution, Abcam) at 4 °C. They were then rinsed three times in PBS and incubated in the dark for 1.5 h with FITC-conjugated goat anti-rabbit IgG diluted 1:400 in PBS, Alexa Fluor 405 anti-rabbit (1:400 dilution, Abcam), Alexa Fluor 488 anti-mouse (1:400 dilution, Abcam), Alexa Fluor 594 anti-rabbit (1:400 dilution, Abcam), or Alexa Fluor 594 anti-goat (1:400 dilution, Abcam) antibody. Finally, the cells and tissue sections were washed three times in PBS and stained with/without DAPI for nuclei staining. The stained sections were wet-mounted with Gel/Mount^TM^ (Biomeda Corp., Foster City, CA, USA), and the images were captured using a confocal laser scanning microscope (Olympus, Tokyo, Japan).

### 4.11. Cytokine Measurement

Analysis of plasma cytokine levels was performed using the Beadlyte^®^ Mouse Multi-Cytokine Detection System (Upstate, Charles, MO, USA) and the Luminex100 luminometer (Luminex Corporation, Austin, TX, USA) according to the manufacturer’s instructions. Fifty-microliter aliquots of plasma were loaded into 96-well microplates with 25 μL of Luminex bead-conjugated anti-mouse multi-cytokine antibody. After 2 h, 25 μL of biotin-conjugated anti-mouse multi-cytokine antibody was added to each well. After incubation for 1.5 h, 25 μL of the streptavidin-phycoerythrin solution was added. After 30 min, 25 μL of stop solution was added. Samples were analyzed using a Luminex 100 luminometer (Luminex, Austin, TX, USA). Quantification of cytokines was performed by regression analysis from a standard curve generated from cytokine standards included in the kit with a lower limit of detection of 10 pg/mL for all cytokines evaluated.

Caudal DRG or spinal cord tissues were homogenized in lysis buffer, and centrifuged at 14,000 rpm (19,300× *g*) for 20 min at 4 °C. The resulting supernatants were then used for further analysis. IL-1β secretion was quantified using ELISA kits (R&D system, Minneapolis, MN, USA) according to the manufacturer’s instructions. The absorbance of the reaction was read on 450 nm with an ELISA reader (Molecular Devices, Sunnyvale, CA, USA).

### 4.12. Electrophysiological Studies

Electrophysiological data were recorded using a patch-clamp amplifier (Axopatch 200, Axon Instruments, Union City, CA, USA). Single-channel currents were digitized using a digital data recorder (VR10, Instrutech, Great Neck, NY, USA) and were stored on videotape. The recorded signals were filtered at 2 kHz using an 8-pole Bessel filter (−3 dB; Frequency Devices, Haverhill, MA, USA) and transferred to a computer using the Digidata 1322A interface (Axon Instruments) at a sampling rate of 20 kHz. Threshold detection of channel openings was set at 50%. The single-channel current tracings shown in the figures were filtered at 2 kHz. In experiments using cell-attached patches and excised patches, pipette and bath solutions contained: 150 mM KCl, 1 mM MgCl_2_, 5 mM EGTA, and 10 mM HEPES (pH = 7.3). The pH was adjusted to the desired values using HCl or KOH. The voltage clamp experiment was performed at room temperature (22–25 °C).

### 4.13. Analysis and Statistics

Real-time PCR data was collected using Light Cycler Software 4.0 (Roche, Mannheim, Germany). LAS-4000 (Fujifilm Corp, Tokyo, Japan), a luminescent image analyzer, was used to capture images of agarose gels and western blots. The bands obtained from PCR and western blot were quantified by Sigma Gel image analysis software (version 1.0, Jandel Scientific, CA, USA) and Quantity One software (version 4.6.3) attached to a GS-800 Calibrated densitometer (Bio-Rad CA, USA). Relative mRNA and protein levels were calculated using referring them to the amount of GAPDH and β-actin, respectively. Student’s *t*-test and one-way ANOVA/Bonferroni test were used to analyze the difference between and among groups, respectively (OriginPro2020, OriginLab Corp. Northampton, MA, USA). Data were presented as the mean ± S.D., and significance was set at *p* < 0.05.

## 5. Conclusions

In conclusion, T9 SCI upregulated TRESK expression in the caudal spinal cord and DRGs. TG_TRESK_ SCI mice were shown to facilitate behavioral recovery and reduce pain sensitivity compared to WT-SCI mice. TG_TRESK_ mice showed lower inflammatory and apoptotic signals than WT mice. These results indicate that TRESK may mainly regulate cell excitability of motor and sensory neurons below the SCI site at acute points.

## 6. Patents

The manufacturing method of a transgenic mouse overexpressing TRESK gene is described in the patent (#10-1036338, A TRANSGENIC ANIMAL OVEREXPRESSING TRESK GENE AND A METHOD FOR PREPARING THE SAME).

## Figures and Tables

**Table 1:** Primer sequences used for RT-PCR and real-time PCR.

Gene Name	Species	GenBank Accession Numbers	Primer Sequences (5′–3′)	Application
*KCNK18*	rat	AY567970	F: CCAGAAGCAGAGGAGAACCC R: CTGCACCAGCATCAATGACA F: ATGCTATATGCACTCTTTGGAAT R: AAGGAGAGCCTGGAACC	RT-PCR Real-time PCR
	mouse		F: ATGTTCCTGGTCCTCACAGA R: TTACCAAGGTAGCGAAACTT	RT-PCR
	TG mouse		F: GGATAGCGGTTTGACTCAGGG R: GCATACAGCATGCACAGGAAC	RT-PCR
*GAPDH*	rat	NM_017008	F: CTAAAGGGCATCCTGGGC R: TTACTCCTTGGAGGCCATG F: CATGGCCTTCCGTGTTC R: CTGCTTCACCACCTTCTT	RT-PCR Real-time PCR

## Supplementary Materials

Supplementary materials can be found at https://www.mdpi.com/1422-0067/21/23/8997/s1. Supplementary Figure S1: Generation of TG_TRESK_ mice.

## Abbreviations

DRG	Dorsal root ganglion
SCI TRESK	Spinal cord injury TWIK (tandem-pore domain weak inward rectifying K^+^)-related spinal cord K^+^ channel
TG_TRESK_	Transgenic mice that overexpress the TRESK gene
