# Supplementary material for: Upregulation of TRESK Channels Contributes to Motor and Sensory Recovery after Spinal Cord Injury
Source: Int J Mol Sci. 2020 Nov 26;21(23):8997. doi: 10.3390/ijms21238997 (PMC7731147; doi:10.3390/ijms21238997)
Supplement: Supplementary file 1 [file ijms-21-08997-s001.pdf]

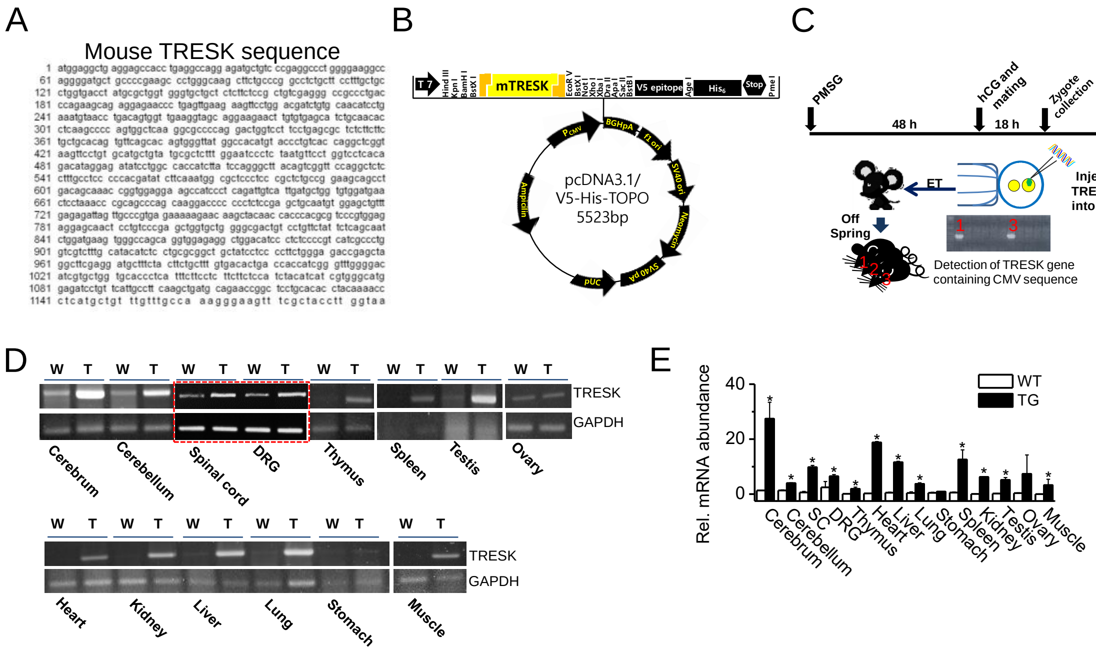

Supplementary Figure 1. Generation of TGTRESK mice (**A**) Mouse TRESK sequence (GenBank accession number, NM_207261) (**B**) Cloning of TRESK into pcDNA3.1/V5-His-TOPO vector. Three restriction enzymes (*Nru*I, *Dra*III, and *Sca*I) were used for elimination of vector sequences. The DNA fragment was used as a transgene for microinjection. (**C**) Injection of the TRESK gene into the male pronucleus of zygotes superovulated by intraperitoneal injection of seven IU of PMSG, followed 48 hours later by an injection of seven IU of hCG. The TRESK gene containing the CMV sequence was detected by PCR with specific primers. (**D**) TRESK mRNA expression was confirmed by semi-quantitative PCR in a variety of organs. The dotted box shows the increase in TRESK expression in spinal cord and DRG obtained from TGTRESK mice. W and T represent wild-type and TGTRESK, respectively. (**E**) Summary of TRESK mRNA levels expressed in the each tissue of TGTRESK mice as judged by real-time PCR. Each bar represents the mean ± SD of five different animals. * $p < 0.05$ compared to corresponding wild type.
